# A mouse model of hepatic encephalopathy: bile duct ligation induces brain ammonia overload, glial cell activation and neuroinflammation

**DOI:** 10.1038/s41598-022-22423-6

**Published:** 2022-10-20

**Authors:** Wouter Claeys, Lien Van Hoecke, Anja Geerts, Hans Van Vlierberghe, Sander Lefere, Griet Van Imschoot, Elien Van Wonterghem, Bart Ghesquière, Roosmarijn E. Vandenbroucke, Christophe Van Steenkiste

**Affiliations:** 1 Barriers in Inflammation, VIB Center for Inflammation Research, VIB, 9052 Ghent, Belgium; 2 Department of Biomedical Molecular Biology, Ghent University, 9000 Ghent, Belgium; 3 Liver Research Center Ghent, Hepatology Research Unit, Department of Internal Medicine and Pediatrics, Ghent University, 9000 Ghent, Belgium; 4 Department of Gastroenterology and Hepatology, Ghent University Hospital, Ghent, Belgium; 5 Metabolomics Expertise Center, Center for Cancer Biology, VIB Center for Cancer Biology, Leuven, Belgium; 6 Metabolomics Expertise Center, Department of Oncology, Katholieke Universiteit Leuven, Leuven, Belgium; 7 Department of Gastroenterology and Hepatology, Antwerp University, Antwerp, Belgium; 8 Department of Gastroenterology and Hepatology, Maria Middelares Hospital, Ghent, Belgium

**Keywords:** Cellular neuroscience, Diseases of the nervous system, Glial biology, Liver diseases

## Abstract

Hepatic encephalopathy (HE) is a common complication of chronic liver disease, characterized by an altered mental state and hyperammonemia. Insight into the brain pathophysiology of HE is limited due to a paucity of well-characterized HE models beyond the rat bile duct ligation (BDL) model. Here, we assess the presence of HE characteristics in the mouse BDL model. We show that BDL in C57Bl/6j mice induces motor dysfunction, progressive liver fibrosis, liver function failure and hyperammonemia, all hallmarks of HE. Swiss mice however fail to replicate the same phenotype, underscoring the importance of careful strain selection. Next, in-depth characterisation of metabolic disturbances in the cerebrospinal fluid of BDL mice shows glutamine accumulation and transient decreases in taurine and choline, indicative of brain ammonia overload. Moreover, mouse BDL induces glial cell dysfunction, namely microglial morphological changes with neuroinflammation and astrocyte reactivity with blood–brain barrier (BBB) disruption. Finally, we identify putative novel mechanisms involved in central HE pathophysiology, like bile acid accumulation and tryptophan–kynurenine pathway alterations. Our study provides the first comprehensive evaluation of a mouse model of HE in chronic liver disease. Additionally, this study further underscores the importance of neuroinflammation in the central effects of chronic liver disease.

## Introduction

Chronic liver disease (CLD) is frequently associated with neuropsychiatric symptoms, ranging from fatigue in primary biliary cirrhosis^1^ to cognitive dysfunction in non-alcoholic fatty liver disease^2^. In cirrhosis, a wide spectrum of neuropsychiatric symptoms, including impairment in cognitive function, motor activity and coordination can be present, called hepatic encephalopathy (HE). HE development is associated with a poor prognosis, with 1-year mortality as high as 50%, and poses an important health care challenge. In addition, current conservative medical treatment is unsatisfactory, whereas liver transplantation, the ultimate treatment for the underlying liver disease, is only available for a subset of patients due to lack of organ donors. While a key role for ammonia and systemic inflammation in HE pathophysiology is obvious^3,4^, profound insight into the mechanisms underpinning brain dysfunction in advanced CLD remains limited, making it hard to design effective brain-targeted treatment options.

Preclinical research in representative models of HE is the first step for getting a better understanding into disease mechanisms and ultimately finding new treatments. The ideal model must fulfil the characteristics described by the International Society of Hepatic Encephalopathy and Nitrogen Metabolism (ISHEN). In summary, a description of a novel HE model should include characterization of underlying liver disease, hyperammonemia, and cognitive/neurological deficits^5^. Ideally the spectrum of neuropsychiatric deficits within this model would span the spectrum of possible symptoms in HE patients, including alterations in consciousness, going from lethargy to somnolence and coma, and motor function alterations, most typically extrapyramidal dysfunction including hypokinesia, bradykinesia and diminished voluntary movement^6^. At present, the rat bile duct ligation (BDL) model has been extensively validated and the most used in HE research^5^. Transgenic animals, which would allow for the disentanglement of brain and liver-specific pathophysiology in HE, are thus far largely restricted to mice. Therefore, a mouse model of HE in CLD would provide an extra tool in HE research. While mouse models of advanced CLD exist^7^, a detailed investigation into peripheral and central HE characteristics is not available for many of these models^5^. Previous studies have shown that BDL in mice can induce cirrhosis^8–12^, behaviour changes^13^ and hyperammonemia^11,14^. Experimental set-up in these reports varies significantly however, preventing recommendations on its generalized usability.

The central nervous system (CNS) microenvironment is tightly regulated by the blood–brain barrier (BBB), and therefore peripheral disturbances do not necessarily translate to the CNS. Due to its proximity, cerebrospinal fluid (CSF) acts as a good approximator of the CNS^15^. HE is characterized by specific metabolic disturbances in the brain compartment. Most prominently, ammonia is metabolized into glutamine through glutamine synthetase, which is exclusively present in astrocytes^16^. Elevated glutamine levels can be detected in CSF of HE patients^15^. In acute liver failure, glutamine attracts water, leading to cytotoxic cerebral edema and intracranial hypertension^17^. In CLD, compensatory decreases in other osmolytes, most notably myo-inositol, prevent development of clinically detectable cerebral edema^18^. In BDL rats, other suggested osmolytes including creatine, taurine and choline decrease, likely as a similar compensatory mechanism^19,20^. Additional metabolic perturbations have been described in both clinical and preclinical HE. Energy metabolism is disturbed in decompensated cirrhosis^21^ and HE patients^15^, possibly by direct influence of ammonia on tricarboxylic acid (TCA) cycle efficiency^22^. Additionally, lactate accumulation secondary to energy failure has been suggested to be involved in cerebral edema in HE^23^. Studies in humans^15^ and animal models of HE^24^ have identified that bile acids accumulate in brains of HE patients and animals, although their precise role in HE pathogenesis is unclear. Finally, in inflammatory conditions, including decompensated cirrhosis, tryptophan metabolization through the tryptophan–kynurenine pathway is augmented^25^, resulting in accumulation of neurotoxic end-products like quinolinic acid. Elevation of tryptophan metabolites in CSF has been suggested as a marker of neuroinflammation^26^. Interestingly, tryptophan degradation products have been found in increased concentrations in CSF of HE patients^15^. However, these findings are often inconsistent between reports or of unknown clinical importance.

Recent concepts of HE pathophysiology have moved away from HE as a pure astrogliopathy and focus more on dysfunction of the multicellular functional base unit of the brain, the neurogliovascular unit (NGVU) (reviewed in^4^). Besides the primordial role of astrocytes in ammonia metabolization, microglia, the resident immune cells of the brain, are of particular interest. Microglial activation has been found in clinical HE^27,28^ and preclinical studies^28,29^. Importantly, microglial morphological changes have already been suggested in BDL mice by one group^13,30,31^, however a standardized quantitative morphological analysis is lacking^32^. Finally, while there is evidence that ammonia itself can cross the BBB^33^, BBB permeability changes have additionally been described in BDL rats, allowing for increased influx of neurotoxins into the brain^34^. High variability in outcomes on the different cells of the NGVU in preclinical HE has been reported. For instance, reports on immunoreactivity of glial fibrillary acidic protein (GFAP), an archetypical marker for astrocyte reactivity, vary with descriptions of increased, decreased or unchanged levels^19,35,36^. Altogether, these data support the need for a thorough temporal evaluation of standard markers of NGVU dysfunction in any new model for HE.

Here, we evaluated and characterized a mouse BDL model that reproduces clinical, metabolic and gliovascular features of HE in CLD and support the clinical relevance of this HE model to use for further neurobiological and intervention studies.

## Materials and methods

### Animals and operational procedures

12–15-week-old male C57Bl/6j wild type mice (Janvier Labs, Le Genest-Saint-Isle, France) and 7-week-old male Swiss wildtype mice (Janvier Labs, Le Genest-Saint-Isle, France) were housed with 14- to 10-h light and dark cycles and with access to food and water in conventional conditions. All experiments comply with the current laws of Belgium (Law of 14. August 1986 related to protection and welfare of animals) and EU directive 2010/63/EU, and were approved by the animal ethics committee of Ghent University (EC2020-051). The bile duct ligation (BDL) and sham procedures were performed under sterile conditions. Under isoflurane inhalation anesthesia, a midline abdominal incision was made and the common bile duct was isolated from the flanking portal vein. The common bile duct was occluded with a double ligature of a non-resorbable suture (Mersilk 5-0) and cut in between ligatures to prevent recanalization. Mice received buprenorphine 0.1 mg/kg IP to prevent postoperative pain and distress^37^. Swiss mice were sacrificed 6 and 8 weeks post-surgery, when cirrhosis developed^8,9^. C57Bl/6j mice were sacrificed on a weekly basis for 4 weeks, starting at 1 week post-surgery. Groups were randomly allocated before study onset. The study is reported in accordance with ARRIVE guidelines.

### Neurobehaviour assessment

Motor function tests were performed at baseline (1 week before model induction) and prior to sampling at each timepoint, more precisely at 7, 14, 21, and 28 days after induction in C57Bl/6j mice and 6 and 8 weeks after induction in Swiss mice. Two tests [difficult beam traversal test and open field test (OFT)] were carried out to gain insights into motor function and exploratory behaviour. In short, in the OFT, mice were allowed to move freely in a clear open-field area (40 × 40 × 40 cm) for 5 min. For the difficult beam traversal, mice were trained to traverse a beam of 1 m with 4 sections of narrowing width (3.5, 2.5, 1.5 and 0.5 cm) placed on top of 2 inverted mouse cages. During the testing phase, a metal grid was placed on top of the beam. Time to traverse the beam was measured. Spatial memory was assessed at the aforementioned timepoints in C57Bl/6j mice. Two tests (novel object recognition (NOR) and T-maze), adapted from^38^, were performed to assess spatial memory. All tests were video recorded. To exclude the existence of olfactory clues, all objects were thoroughly cleaned with 20% ethanol after each trial. Analysis of OFT and NOR test was performed using Noldus Ethovision XT 15 software (Noldus). Detailed outline of procedures is available in supplementary materials and methods.

### Plasma biomarker measurement

After sedation with ketamine (87.5 mg/kg) and xylazine (12.5 mg/kg), whole blood was withdrawn using heart puncture. For ammonia measurement, EDTA plasma samples were immediately put on ice and analysed on the Architect c16000 (Abbott) within 1 h after sample isolation^5^. Plasma levels of ALT, AST, albumin and bilirubin were assessed using the Architect c16000 (Abbott).

### CSF isolation

Mice were sacrificed using an overdose of ketamine (87.5 mg/kg) and xylazine (12.5 mg/kg). After disappearance of the paw and tail reflexes, cerebrospinal fluid (CSF) was obtained from the fourth ventricle via the cisterna magna puncture method as described previously^39^. Briefly, borosilicate glass capillary tubes (Sutter Instruments; B100-75-15) were used to pull needles on the Sutter P-87 flaming micropipette puller (pressure 330 Pa, heat index 300). The incision site was sterilized with 70% ethanol. The cisterna magna was exposed by dissecting skin and muscle tissue on the posterior side of the skull. The head of the mouse was mounted at an angle of 135°, and CSF was collected from the fourth ventricle by puncturing the cisterna magna using the capillary needles. Samples were centrifuged for 5 min at 300*g* at 4 °C to assess blood contamination. For initial experiments, samples were pooled up to a total volume of 10 µl. All other samples were not pooled. After centrifugation, CSF samples were snap frozen in liquid nitrogen and stored at -80 °C until further usage.

### Targeted metabolomics

The target compounds were purchased as purified standards, prior to the analysis, these standards were run to obtain both retention time info as well as the accurate mass of the target metabolites. 10 μL of each sample was extracted in 190 μL 80% MeOH containing 2 μmol/L of internal standard (d27 myristic acid). 10 μL of each sample was loaded into a Dionex UltiMate 3000 LC System (Thermo Scientific Bremen, Germany) equipped with a C-18 column (Acquity UPLC-HSS T3 1. 8 μm; 2.1 × 150 mm, Waters) coupled to a Q Exactive Orbitrap mass spectrometer (Thermo Scientific) operating in negative ion mode. A step gradient was carried out using solvent A (10 mM TBA and 15 mM acetic acid) and solvent B (100% methanol). The gradient started with 5% of solvent B and 95% solvent A and remained at 5% B until 2 min post injection. A linear gradient to 37% B was carried out until 7 min and increased to 41% until 14 min. Between 14 and 26 min the gradient increased to 95% of B and remained at 95% B for 4 min. At 30 min the gradient returned to 5% B. The chromatography was stopped at 40 min. The flow was kept constant at 0.25 mL/min at the column was placed at 40 °C throughout the analysis. The MS operated in full scan mode (m/z range: [70.0000–1050.0000]) using a spray voltage of 4.80 kV, capillary temperature of 300 °C, sheath gas at 40.0, auxiliary gas at 10.0. The AGC target was set at 3.0E + 006 using a resolution of 140,000, with a maximum IT fill time of 512 ms. Area of the peaks from target metabolites were integrated by El-Maven (Elucidata).

### Immunostainings and microglia and astrocyte 3D reconstruction

For immunostainings performed on brain and liver sections, mice were transcardially perfused with ice-cold 4% PFA in PBS. Subsequently, liver and brain tissue (carefully extracted from the skull) were post-fixed overnight in 4% PFA in PBS. The left brain hemisphere was embedded in 5% agarose covered with 0.01% sodium azide and stored at 4 °C until further use. After dehydration, liver samples and the right brain hemispheres were embedded in paraffin at room temperature (RT) until further use. For cryosections, right brain hemispheres were embedded in Frozen Section Medium (Thermo Scientific) immediately after transcardial perfusion in cryomolds (Sakura) that were frozen on dry ice and stored at − 80 °C until further use.

Five µm liver tissue sections were cut (HM 340 E, Thermo Fischer Scientific) and stained with haematoxylin–eosin (Klinipath) or 0.1% Sirius Red. Sections were imaged using Zeiss Axioscan Z.1 (10 × magnification, Zeiss, Germany). All images were stained simultaneously and evaluated in a blinded manner. Bile infarcts and Sirius Red area fractions were quantified using ImageJ (version 1.53c, National Institutes of Health). Images of at least 10 random low power fields were analyzed per liver section. Fibrosis was quantified as a percentage Sirius red stained area relative to the total section area and compared to levels of sham mice. Bile infarcts were quantified on H&E sections as a percentage necrotic are relative to the total section area.

The brains were cut into 5 μm paraffin sections (HM 340 E, Thermo Scientific) or 20 μm cryosections (CryoStat NX70, Thermo Scientific). For immunofluorescence staining, sections were permeabilized in PBS containing 0.5% Triton X-100. Following blocking with goat immunomix (GIM) (5% goat serum, 0.5% Triton X-100 in PBS) at RT for 1 h, sections were incubated with primary antibodies [anti-IBA1 (1/500, Wako Chemicals, 019-19741), anti-OCLN (1/100, Invitrogen, 33-1500), anti-ZO-1 (1500, Invitrogen, 617300)] in GIM at 4 °C overnight. After washing with PBS, sections were stained with fluorophore-conjugated secondary antibodies in PBS at RT for 2 h. Counterstaining was done with DAPI 1/1000 in PBS. Confocal images were taken with a Zeiss LSM 780 (Zeiss, Germany), using a Plan-Apochromat 40 × 1.3 oil DIC UV-IR M27 objective (OCLN, ZO-1) or a Plan-Apochromat 25 × 0.8 Imm Korr DIC M27 objective (IBA1). IBA1 + cells were manually counted, IBA1 + area fraction was determined using ImageJ (version 1.53c, National Institutes of Health).

For 3D reconstruction of microglia and astrocytes, 50 μm thick vibratome sagittal brain sections were permeabilized in PBS containing 0.5% Triton X-100. Following blocking with GIM at RT for 1 h, sections were incubated with primary antibodies (anti-IBA1 (1/500, Wako Chemicals, 019-19741), anti-GFAP (1/1000, Agilent, Z033429-2) in GIM at 4 °C overnight. After washing with PBS, sections were stained with fluorophore-conjugated secondary antibodies in PBS at RT for 2 h. Z-stack images were taken with a Zeiss LSM 780 (Zeiss, Germany), using a Plan-Apochromat 40 × 1.3 oil DIC UV-IR M27 objective. Maximum intensity projection images were produced using ImageJ (version 1.53c, National Institutes of Health). The 3D reconstructions and measurements were performed by filament tracing algorithm from Imaris software (Bitplane). Detailed description of primary and secondary antibodies used can be found in Supplementary Table 1.

### Tissue isolation

For RNA and protein analysis, mice were perfused with a D-PBS/heparin [0.2% heparin (5000 IU/mL, Wockhardt)]. Selected brain regions (prefrontal cortex, hippocampus, cerebellum, rest brain) and liver tissue were isolated, snap frozen in liquid nitrogen and stored at − 80 °C until analysis. Liver samples for RNA analyses were submerged in an excess of RNA Later (Thermo Fischer Scientific) and kept overnight at 4 °C, after which they were transferred to − 80 °C until further analysis.

### RNA isolation and real-time qPCR

RNA was isolated using the Aurum total RNA Mini Kit (Bio-Rad), according to the manufacturer’s instructions. RNA concentration and purity were determined spectrophotometrically using the Nanodrop ND-1000 (Nanodrop Technologies, Thermo Scientific), and cDNA was synthesized with the SensiFAST™ cDNA Synthesis Kit (Bioline). Real time-qPCR was performed with the Light Cycler 480 system (Roche) using SensiFast SYBR No-Rox (Bio-Line). Expression levels were normalized to the expression of all stable (at least 2) reference genes, determined using the geNorm Housekeeping Gene Selection Software^40^. Sequences of forward and reverse primers can be found in Supplementary Table 2.

### Cytokine measurement

Prefrontal cortex samples were homogenized with lysis buffer [RIPA in PBS supplemented with complete protease inhibitor (Thermo Scientific)] using a Tissuelyzer II (Qiagen, 20 Hz for 5 min). Supernatant was collected by centrifugation at 20,000*g* in a microcentrifuge at 4 °C for 15 min. Protein concentration of the samples was measured using the Pierce BCA Protein Assay Kit (Thermo Scientific). Cytokines in plasma (IL-1β, IL-6, TNF) and prefrontal cortex lysates (IL-1β, IL-6, TNF, MCP-1, MIP-1α, MIP-1β, IL-4, IL-10, IL-17, RANTES) were assessed using the Bio-Plex cytokine assay (Bio-Rad) according to the manufacturer’s instructions.

### Analysis of BBB integrity

Fifteen minutes before sacrifice, mice (n = 5–8 per group) were intravenously (iv) injected with 250 mg/kg FITC-labeled dextran (4 kDa, Sigma). Mice were perfused with D-PBS/heparin (0.2% heparin (5000 IU/mL, Wockhardt) to remove blood. Adequate perfusion was checked visually. Brain samples were lysed mechanically and subsequently in formamide at 37 °C in the dark overnight. To measure BBB leakage, weight-corrected tissue fluorescence was assessed (λ_ex_/λ_em_ = 485/520 nm) using the FLUOstar omega (Isogen LifeScience).

### Statistical analysis

All data are represented as mean ± SEM. Differences were considered significant at p < 0.05. For comparison of two groups, unpaired *t* test or Mann–Whitney test were used based on normality testing of data. For comparison of multiple groups, significance was determined using one-way ANOVA with Dunnett post-hoc testing or Kruskal–Wallis with Dunn post-hoc testing, based on normality distribution of residuals. For data with both time and treatment factors, 2-way ANOVA with Sidak post-hoc testing was performed. Correlation was assessed using Spearman correlation testing. All testing was two-sided. Graphpad 9.2 (LaJolla, California) was used for all statistical analyses.

### Institutional review board statement

The study was conducted according to the guidelines of the Declaration of Helsinki and approved by the Institutional Ethics Committee of Ghent University (EC2020-051/17 Nov 2020).

## Results

### BDL in C57Bl/6j mice reproduces core characteristics of HE

**Figure 1:**
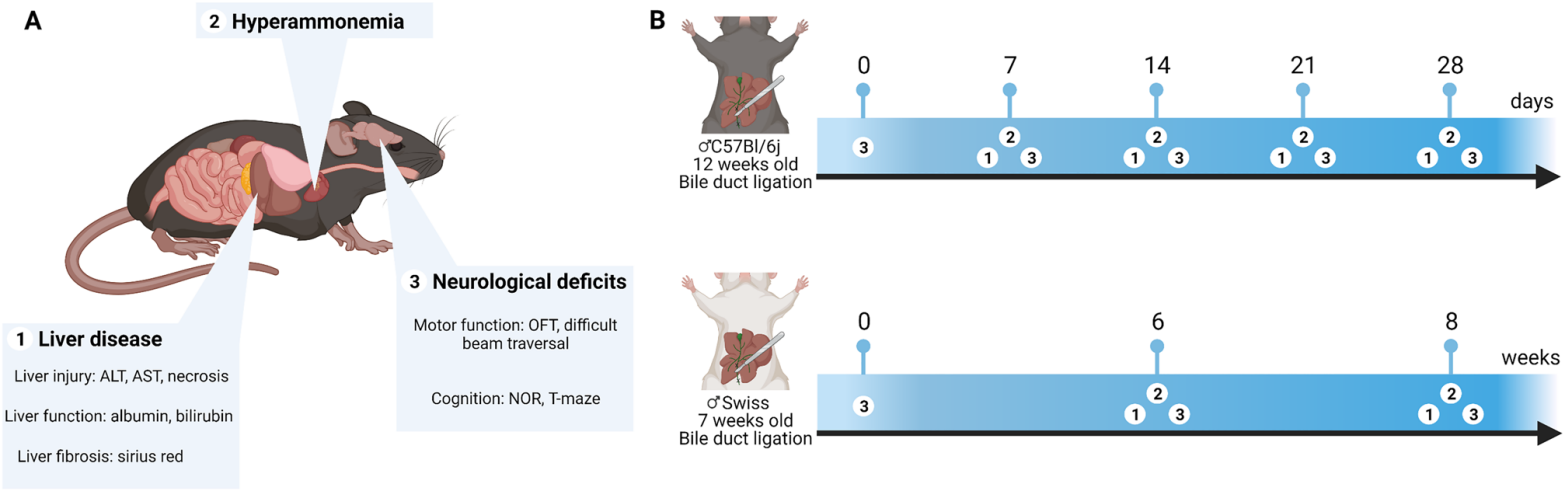
(**A**) Selected outcome measures to determine underlying liver disease, hyperammonemia and neurological deficits according to ISHEN guidelines. (**B**) Timeline detailing timing of outcome measure assessment after BDL in both C57Bl/6j and Swiss mice. *BDL* bile duct ligation, *ISHEN* International society for hepatic encephalopathy and nitrogen metabolism.

As described above, it is essential to evaluate a suitable hepatic encephalopathy (HE) model in chronic liver disease (CLD) based on the International Society of Hepatic Encephalopathy and Nitrogen Metabolism (ISHEN) guidelines. This includes assessment of liver injury, hyperammonemia and neurological, behavioural or motor impairment. Detailed experimental design can be found in Fig. 1. As liver disease development is impacted heavily by the strain^41^, both C57Bl/6j and Swiss mice were used. We previously established that BDL induces cirrhosis in Swiss mice from 6 weeks after induction, while prolonging the model to 10 weeks induces quasi 100% spontaneous mortality^8,9^. Therefore, analyses were carried out 6 and 8 weeks after induction. In C57Bl/6j mice however, earlier mortality has been described^12^, while indices of liver disease do not progress significantly beyond 28 days after induction, which was therefore chosen as a study endpoint^42,43^. Spontaneous mortality was comparable between strains at study endpoint (Fig. S1).

**Figure 2:**
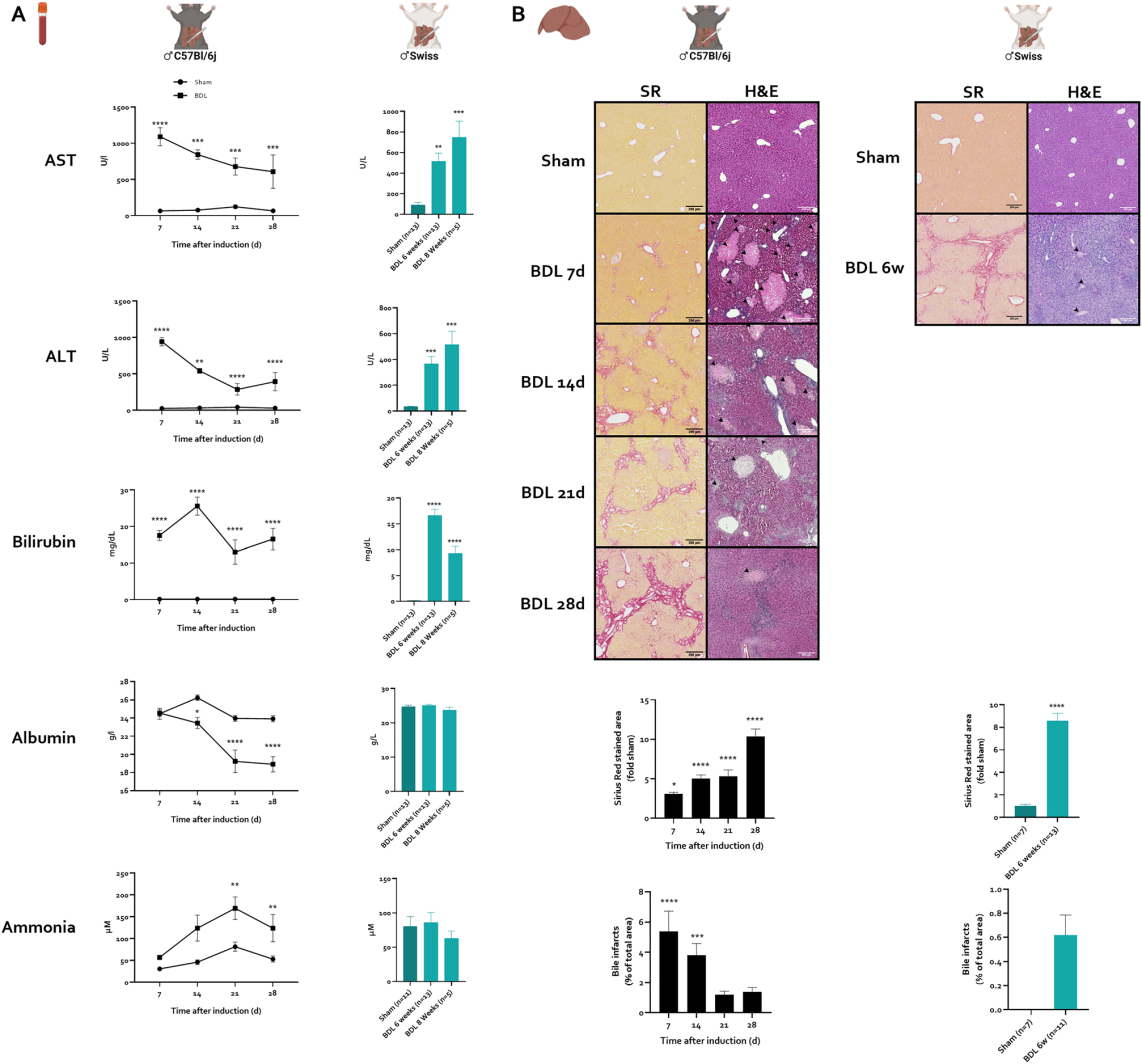
Evolution of liver disease in C57Bl/6j and Swiss mice after chronic BDL. C57Bl/6j mice were subjected to sham or BDL for 7, 14, 21 and 28 days. Swiss mice were subjected to sham or BDL for 6 and 8 weeks. (**A**) Plasma levels of AST, ALT, bilirubin, albumin and ammonia at different timepoints after sham (C57Bl/6j mice: n = 5–15/timepoint; Swiss mice: n = 13) or BDL (C57Bl/6j mice: n = 6–9/timepoint; Swiss mice: n = 5–13/timepoint) induction. (**B**) Representative H&E and Sirius Red stained images after sham and BDL induction. Quantification of the fibrotic area on Sirius Red stained liver sections of sham (C57Bl/6j mice: n = 5/timepoint; Swiss mice: n = 7) or BDL (C57Bl/6j mice: n = 5/timepoint; Swiss mice: n = 13) mice at different timepoints. Quantification of biliary infarcts on H&E-stained liver sections of sham (C57Bl/6j mice: n = 5/timepoint; Swiss mice: n = 7) or BDL (C57Bl/6j mice: n = 5/timepoint; Swiss mice: n = 11) mice at different timepoints. Data are represented as means ± SEM. *p < 0.05, **p < 0.01, ***p < 0.001, ****p < 0.0001 vs. sham control. Arrowheads indicate biliary infarcts. Scale bars represent 200 µm. *BDL* bile duct ligation.

The bile duct ligation (BDL) model is a typical model for induction of secondary biliary injury^44^. Liver injury was assessed through plasma AST and ALT levels. In C57Bl/6j mice, plasma ALT and AST rise early, peaking at 7 days post-surgery. Transaminase levels decrease over time but remain elevated compared to sham mice in all assessed timepoints (Fig. 2A). In Swiss mice, both ALT and AST levels are elevated at 6 weeks post-surgery and continue to rise 8 weeks after surgery compared to sham controls (Fig. 2A). Liver function was assessed through evaluation of circulating levels of bilirubin and albumin. Bilirubin is consistently elevated at all timepoints in both strains (Fig. 2A). Albumin levels, a marker of liver synthetic capacity, are significantly decreased from 14 days onward (Fig. 2A) in C57Bl/6j mice but remain unchanged in Swiss mice after BDL surgery (Fig. 2A).

Secondly, plasma ammonia levels, the hallmark of HE, were determined. Plasma ammonia levels progressively increase in C57Bl/6j BDL mice, peaking 21 days after induction (Fig. 2A; 169.5 vs. 81.58 µM), after which levels again slightly decrease at 28 days (Fig. 2A). In Swiss mice, plasma ammonia levels remain unchanged after BDL surgery compared to sham controls at both timepoints (Fig. 2A).

Histological evaluation of liver fibrosis, a hallmark of CLD, reveals significant fibrosis development after BDL in both strains. In C57Bl/6j mice, increased Sirius red positive area is already detectable 7 days after induction and progressively increases over all assessed timepoints (Fig. 2B). All C57Bl6/j BDL mice exhibit portal-portal bridging at the 28-day timepoint (Fig. 2B). In Swiss mice, extensive fibrosis with occasional nodular changes indicative of cirrhosis is detectable 6 weeks after induction (Fig. 2B). After BDL, clusters of injured hepatocytes called bile infarcts develop secondary to cholestasis^42,45^. Bile infarct area, an alternative marker of liver injury, peaks 7 days after BDL in C57Bl/6j mice and decreases over later timepoints, with low infarct area at 28 days post-surgery, not significantly higher than sham control animals. In Swiss mice, while bile infarctions were detectable, infarct area is low (Fig. 2B).

**Figure 3:**
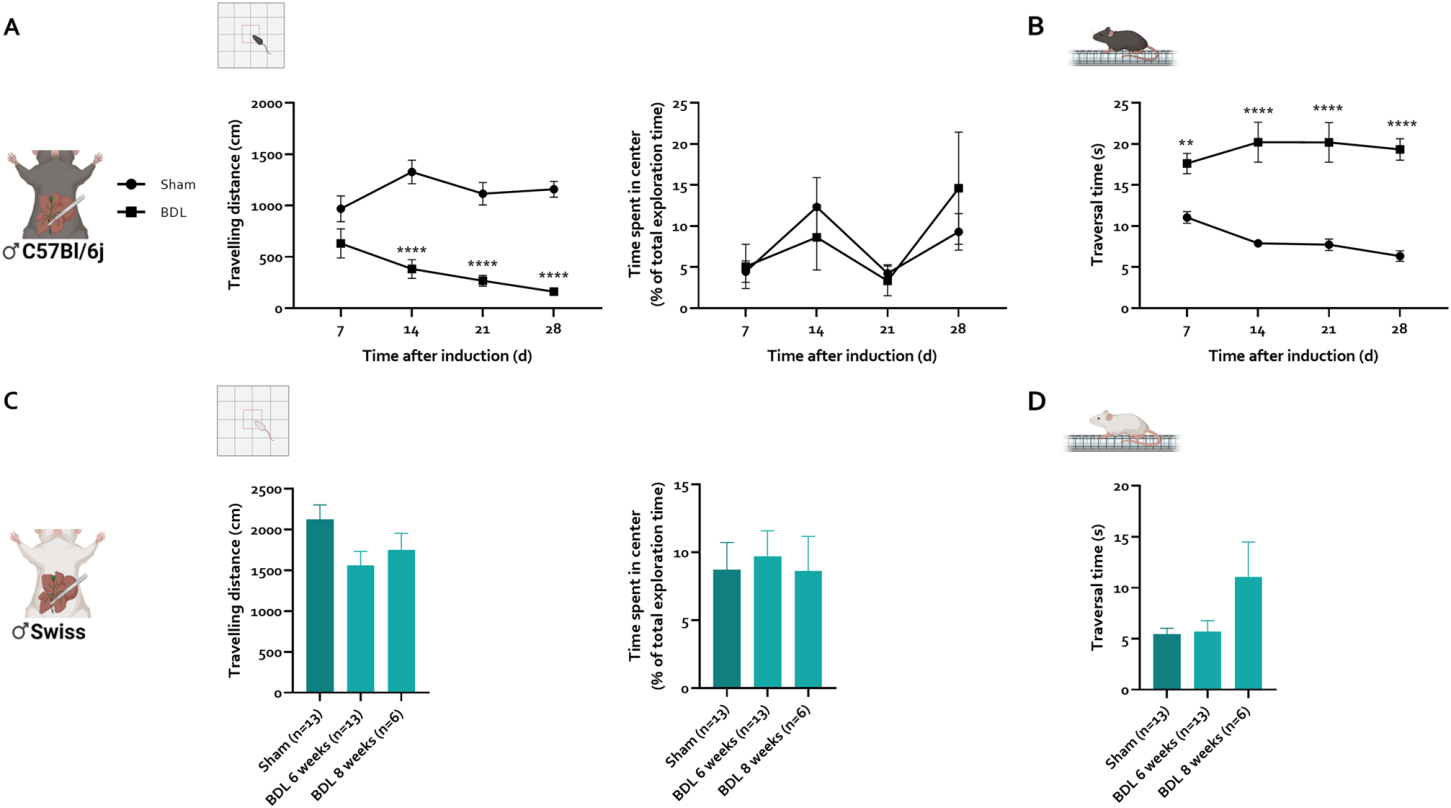
Evolution of behaviour changes after chronic BDL in C57Bl/6j and Swiss mice. C57Bl/6j mice were subjected to sham (n = 15) and BDL (n = 17) for 28 days. Swiss mice were subjected to sham (n = 13) and BDL for 6 (n = 13) or 8 (n = 6) weeks. (**A**) Travelling distance, time spent in the center of the open field at different timepoints after sham/BDL inductions in C57Bl/6j (**A**) and Swiss (**C**) mice. Average traversal time over the difficult beam at different timepoints after sham/BDL inductions in C57Bl/6j (**B**) and Swiss (**D**) mice. Data are represented as means ± SEM. *p < 0.05, **p < 0.01, ***p < 0.001, ****p < 0.0001 vs. sham control. *BDL* bile duct ligation.

To assess neurobehavioural abnormalities consistent with HE, standard motor function and cognition tests were performed. The open field test (OFT) is used to assess general motor activity and anxiety levels. In C57Bl/6j mice, travelling distance in the OFT is significantly reduced from 14 days after BDL induction (− 71% vs. sham) and further decreases in later timepoints (Fig. 3A). The time spent in the center of the open field is not different between sham controls and BDL mice at any given timepoint, indicating that anxiety is not a factor (Fig. 3A). The difficult beam traversal test was performed as a measure of balance. Performance in the difficult beam traversal test is disturbed even earlier, with significantly slower traversal time in BDL animals compared to sham controls after 7 days (+ 60% vs. sham). Traversal time remains significantly elongated over all assessed timepoints (Fig. 3B). In Swiss mice, performance in the OFT is slightly decreased 6 and 8 weeks after BDL induction, not reaching statistical significance (Fig. 3C). Additionally, performance in the difficult beam traversal is not significantly altered 6 or 8 weeks after BDL in this strain (Fig. 3D). Novel object recognition (NOR) and T-maze tests were used as measures of spatial memory. However, due to the severe motor function alterations in C57Bl/6j BDL mice, most mice failed to meet minimum exploration criteria during both tests. We therefore concluded that cognition testing is confounded and cannot be assessed in a reliable way (data not shown).

### BDL induces systemic inflammation and the neurometabolic signature of HE

While the recent ISHEN guidelines stress the need for characterization of the features detailed above^5^, other factors not included there have been suggested to be instrumental in disease development^46^ and should therefore be included in a thorough model characterization.

**Figure 4:**
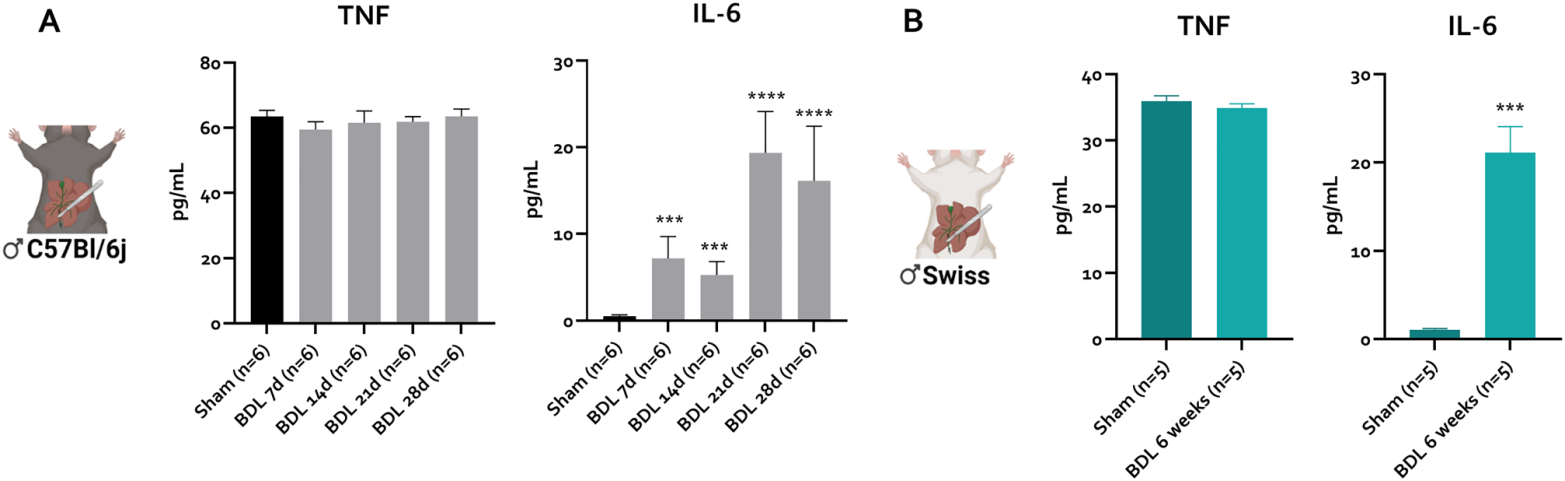
Plasma cytokine levels post BDL in C57Bl/6j and Swiss mice. Plasma levels of IL-6 and TNF in sham (C57Bl/6j: n = 6; Swiss: n = 5) controls and post-BDL (C57Bl/6j: n = 6/timepoint; Swiss: n = 5). Data are represented as means ± SEM. *p < 0.05, **p < 0.01, ***p < 0.001, ****p < 0.0001 vs. sham control. *BDL* bile duct ligation, *IL* interleukin, *TNF* tumor necrosis factor.

According to current knowledge, hyperammonemia synergizes with systemic inflammation to induce clinical HE^4^. We assessed circulating levels of IL-6, IL-1β and TNF as markers of systemic inflammation. In both strains, IL-1β cannot be detected in plasma and circulating TNF levels are not significantly altered after BDL (Fig. 4A,B). In both strains, IL-6 is elevated after BDL (Fig. 4A,B). In C57Bl/6j mice, this elevation compared to sham controls can already be observed 7 days after BDL (Fig. 4A) and further increases in later timepoints (Fig. 4A).

**Figure 5:**
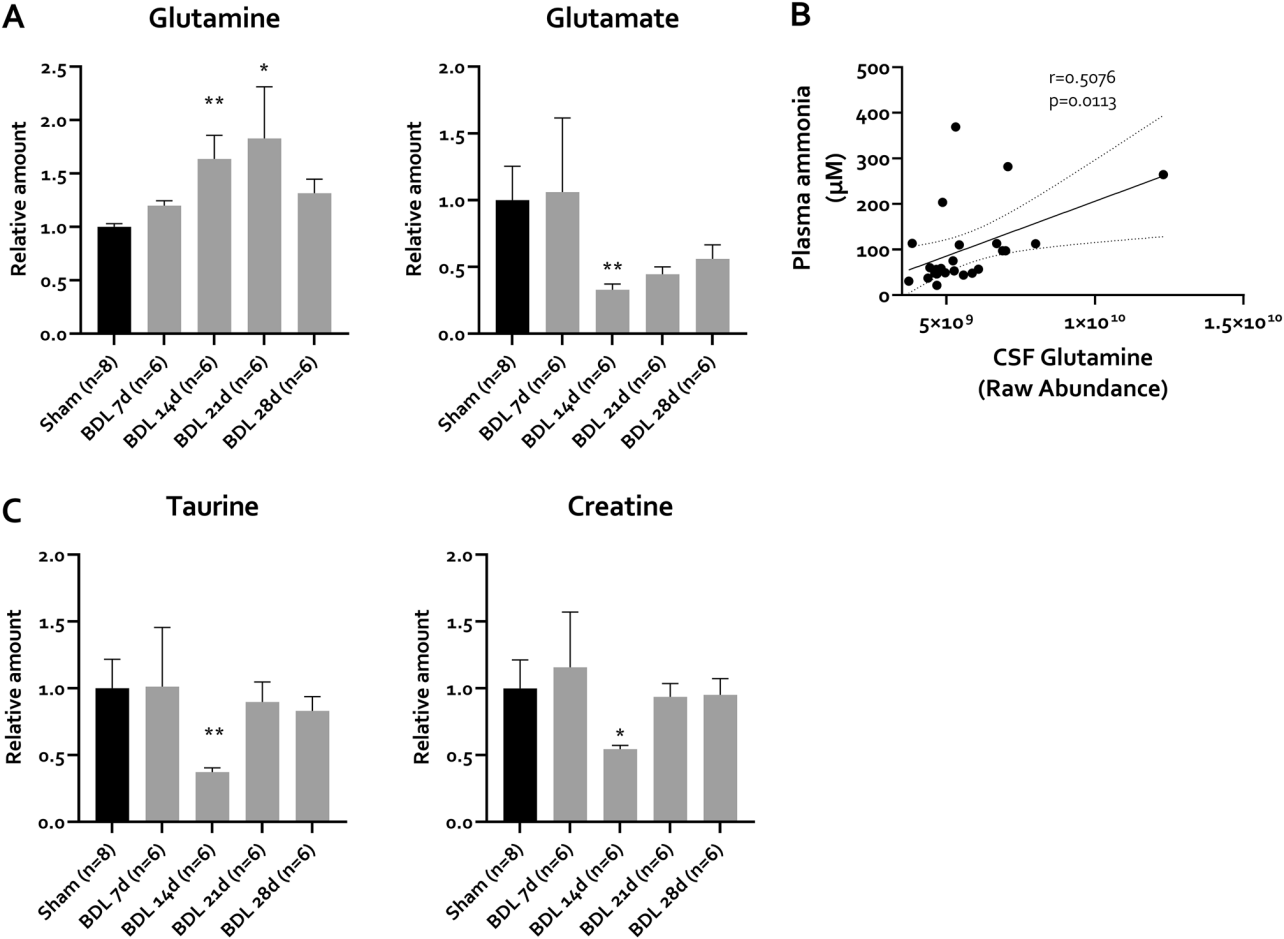
Ammonia-related metabolites in the cerebrospinal fluid (CSF) of C57Bl/6j mice. (**A**–**C**) Relative CSF levels of (**A**) glutamine, glutamate and (**C**) osmolytes taurine and creatine in sham controls (n = 8) and BDL (n = 6/timepoint) animals at different timepoints. (**B**) Spearman correlation between CSF glutamine and plasma ammonia. Spearman correlation coefficient (r) and corresponding p-value are shown. Values are represented as mean ± SEM. *p < 0.05, **p < 0.01. *BDL* bile duct ligation, *CSF* cerebrospinal fluid.

As only C57Bl/6j mice develop hyperammonemia and behaviour changes, further experiments were only performed with this strain. To assess whether peripheral changes are associated with central nervous system (CNS) alterations suggestive of HE, we assessed selected key metabolites in the cerebrospinal fluid (CSF) of BDL and sham animals. In the brain, ammonia is metabolized into glutamine, consuming glutamate^16^. In chronic HE, this is often accompanied by decreases in other osmotically active molecules^19^. In a preliminary experiment, targeted CSF metabolomics revealed a significant increase in glutamine in CSF of BDL mice compared to sham controls, 28 days after induction. Moreover, there was a trend towards decreasing glutamate and creatine, as well as a significant decrease in taurine levels (Fig. S2). Evaluation of model kinetics confirms these observations and reveals a progressive increase in CSF glutamine levels of BDL mice, reaching significance from 14 days onward (Fig. 5A). At this timepoint, glutamate, taurine and creatine are decreased, consistent with a compensatory osmotic response (Fig. 5A,B). While glutamate levels remain decreased over the full time course, albeit not significantly compared to sham controls, levels of taurine and creatine normalize from 21 days onward. To assess whether metabolic changes in the brain were linked with the periphery, correlation analysis was performed. This reveals that plasma ammonia levels correlate positively with CSF glutamine levels (Fig. 5C).

### Neurometabolic profiling of BDL mice

**Figure 6:**
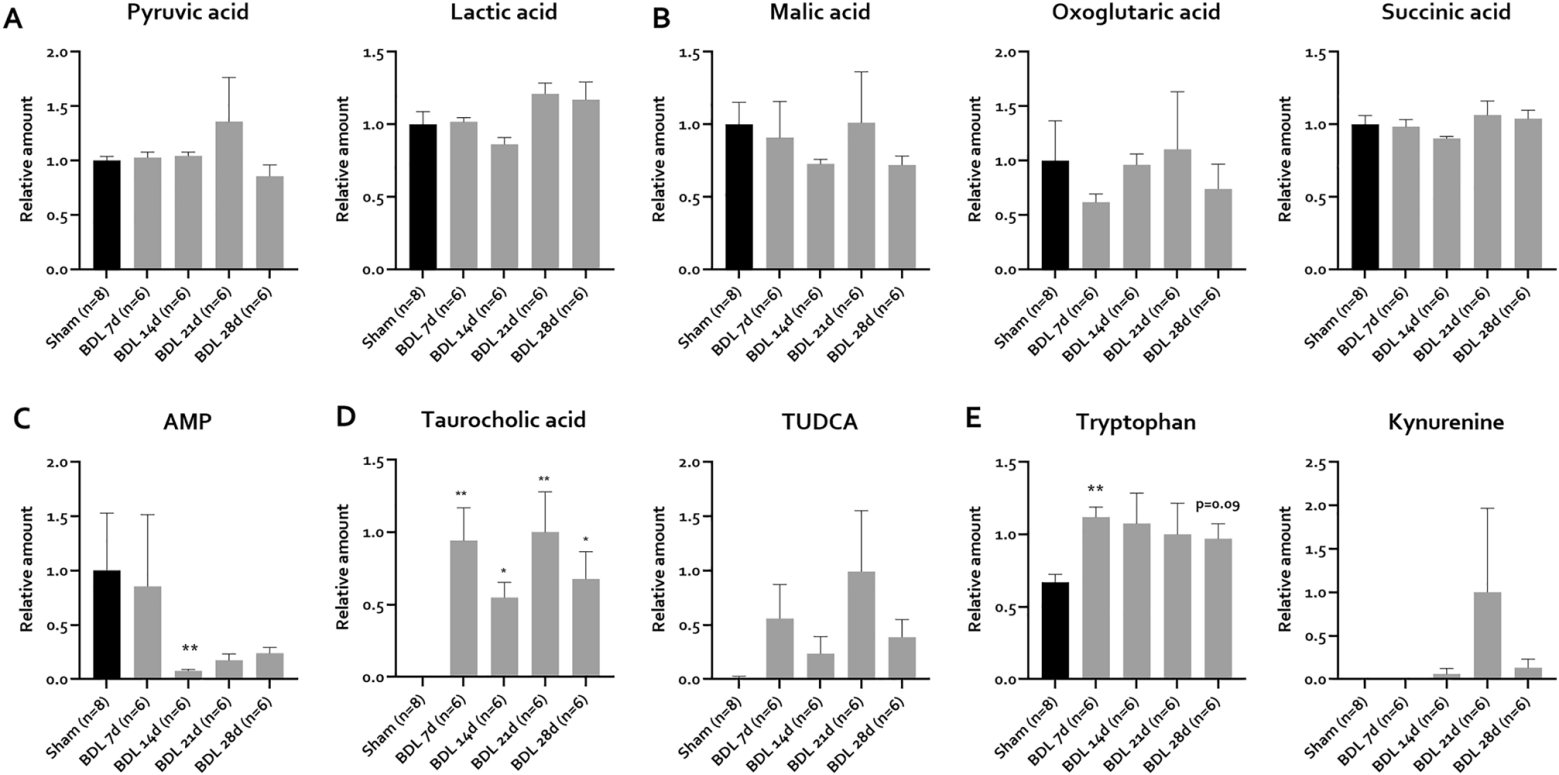
Energy markers, bile acids and tryptophan metabolites in CSF post BDL in C57Bl/6j mice. (**A**–**E**) Relative CSF levels of (**A**) glycolysis intermediates and end products, (**B**) TCA cycle intermediates, (**C**) AMP, (**D**) bile acids and (**E**) tryptophan metabolites in sham controls (n = 8) and BDL animals (n = 6/timepoint) at different timepoints. Values are represented as mean ± SEM. *p < 0.05, **p < 0.01. *BDL* bile duct ligation, *CSF* cerebrospinal fluid, *TCA* tricarboxylic acid.

Several metabolic pathways implicated in HE pathogenesis were assessed in BDL mice. To investigate energy metabolism in BDL mice, targeted metabolomic analysis of energy status and glycolysis and TCA cycle intermediates was performed. Lactate, pyruvate, malate, oxoglutarate, succinate and AMP can be detected in CSF. On the other hand ATP, ADP and oxalacetic acid are not detectable in any sample. Lactate and pyruvate levels are not significantly changed after BDL surgery (Fig. 6A). Also, the TCA cycle intermediates malic acid, succinic acid and oxoglutaric acid are not significantly altered by BDL surgery (Fig. 6B). Interestingly, AMP is depleted 14 days after BDL (Fig. 6C) and a similar, non-significant trend continues over later timepoints.

With regards to bile acids, levels selected bile acids implicated in HE pathophysiology were assessed^47,48^. All bile acids are undetectable in sham CSF samples. Only two bile acids, namely taurocholic acid and tauroursodeoxycholic acid are detectable from 7 days onwards in BDL mice, with fluctuating but consistently elevated concentrations over time (Fig. 6D).

In the context of (neuro)inflammation, extrahepatic tryptophan metabolism through the kynurenine pathway is amplified, leading to elevated concentrations of tryptophan–kynurenine pathway metabolites like the neurotoxic quinolinic acid^25^. To investigate central tryptophan–kynurenine metabolism, tryptophan and related metabolites were evaluated in the CSF. Interestingly, tryptophan is found to be increased compared to sham controls in CSF 7 days post BDL. This trend continues over later timepoints, however failing to reach statistical significance. Interestingly, while kynurenic and quinolinic acid can’t be detected in any of the CSF samples, kynurenine is undetectable in sham animals, but present in some samples at later timepoints after BDL (n = 1 at 14 days, n = 4 at 21 days and n = 3 at 28 days), consistent with progressive neuroinflammation (Fig. 6E).

Ammonia can induce oxidative stress^49^. However oxidative stress markers have not universally been detected in HE brains^50,51^. Targeted metabolomics reveals no significant difference in CSF concentrations of (oxidized) glutathione and CSSG (Fig. S3). Other markers NADP and NADPH cannot be detected in the CSF of BDL or sham animals.

### Characterisation of gliovascular changes in BDL mice

HE can be described as a dysfunction of the functional base unit of the brain, the neurogliovascular unit (NGVU) (reviewed in^4^). Neuronal support cells, and in particular astrocytes and microglia, seem to play an instrumental role in NGVU dysfunction in HE. We therefore characterized morphological and functional changes in these cell types in the BDL model.

**Figure 7:**
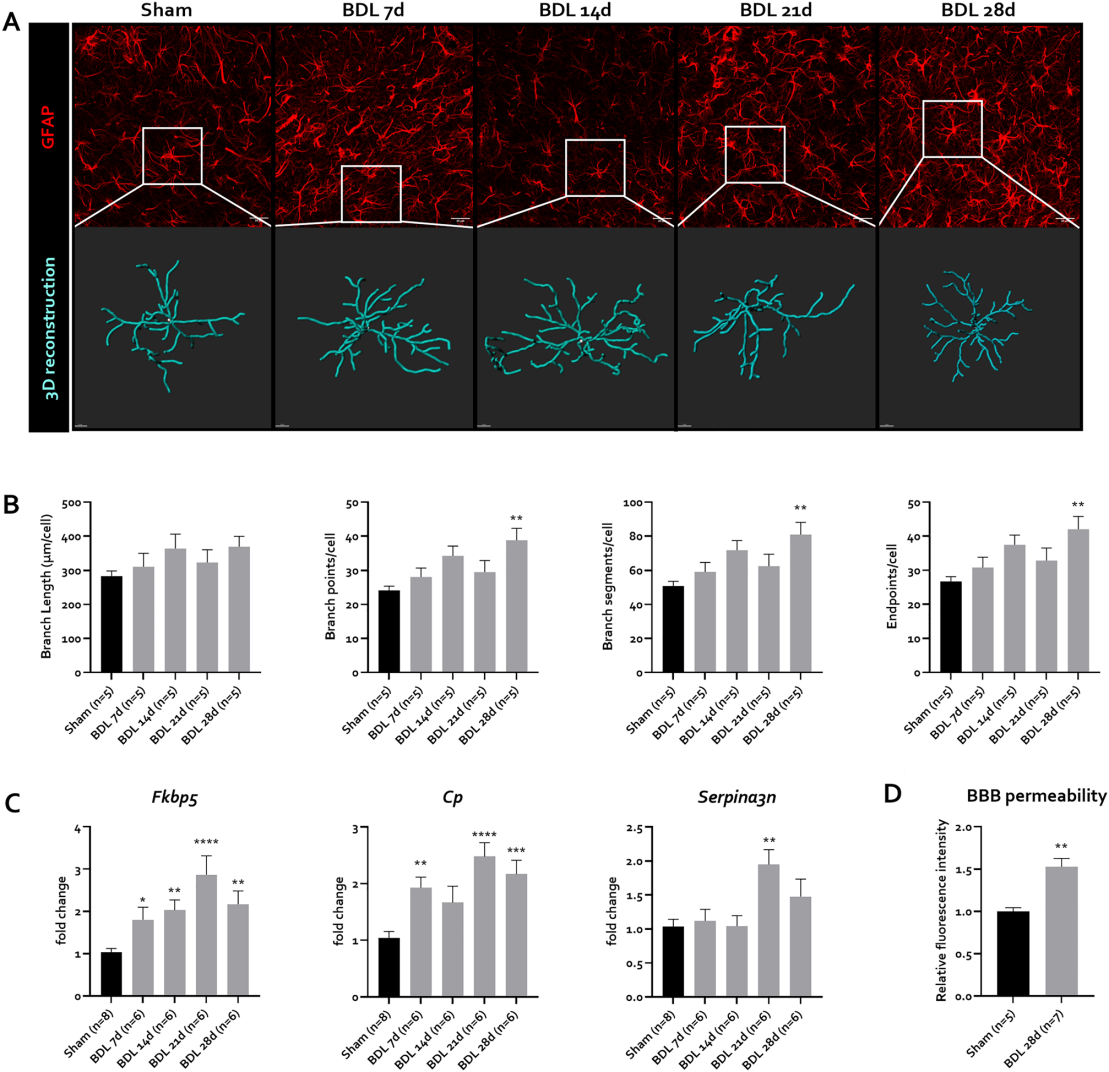
Astrocyte reactivity and function after BDL in C57Bl/6j mice. (**A**) Representative confocal maximum intensity projection images of GFAP positive astrocytes and magnified 3D reconstructed astrocytes in the CA3 region of the hippocampus at different timepoints after BDL. (**B**) Quantification of 3D reconstructed astrocytes, showing branch length, number of branch points, branch segments and endpoints per cell in sham (n = 5) or BDL animals (n = 5/timepoint) at different timepoints after BDL. (**C**) Expression levels of astrocyte reactivity markers Fkbp5, Cp and Serpina3n in sham (n = 8) and BDL (n = 6/timepoint) mice at different timepoints after BDL. (**D**) Blood–brain barrier (BBB) permeability assessment through measurement of brain tissue fluorescence after iv injection of 4 kDa FITC-Dextran in sham (n = 5) and BDL (n = 7), 28 days after induction. All data are represented as mean ± SEM. *p < 0.05, **p < 0.01, ***p < 0.001, ****p < 0.0001. Scale bar GFAP = 20 µm. Scale bar 3D reconstruction = 5 µm. *BBB* blood–brain barrier, *BDL* bile duct ligation, *Cp* ceruloplasmin (Cp), *Fkbp5* FK506 binding protein 5 (Fkbp5), *GFAP* glial fibrillary acidic protein, *Serpina3n* serpin family A member 3.

In response to a pathological CNS microenvironment, astrocytes undergo remodelling known as ‘astrocyte reactivity’, classically assessed through increased glial fibrillary acidic protein (GFAP) immunoreactivity. Based on recent guidelines, molecular and functional readouts were additionally assessed to characterize this astrocyte reactivity^52^. 3D reconstruction of GFAP positive astrocytes in BDL mice reveal a subtle but stepwise increase in arborization, with significantly increased branching (branch segments and branch points) and terminal points 28 days after BDL induction (Fig. 7A,B). Additionally, expression of recently identified astrocyte reactivity marker genes FK506 binding protein 5 (*Fkbp5*), ceruloplasmin (*Cp*) and serpin family A member 3 (*Serpina3n*)^53^ mirrors the evolution of morphological changes, consistent with astrocyte reactivity (Fig. 7C). As a functional read-out, BBB permeability is increased 28 days after induction, as evident from increased 4 kDa FITC-Dextran signal in brain tissue of BDL mice (Fig. 7D). At that timepoint, we are unable to find any significant changes in staining pattern of tight junction components zonula occludens-1 (ZO-1) and occludin (OCLN) (Fig. S4A,B). While *Ocln* gene expression levels show a limited but significant increase (Fig. S4B), we do not find altered gene expression levels of genes *Zo1*, *Cldn5* and *Cdh1* 28 days after BDL (Fig. S4B). This suggests that the observed loss of BBB permeability is not mediated by major tight junction relocalization or altered gene expression of tight junction components.

**Figure 8:**
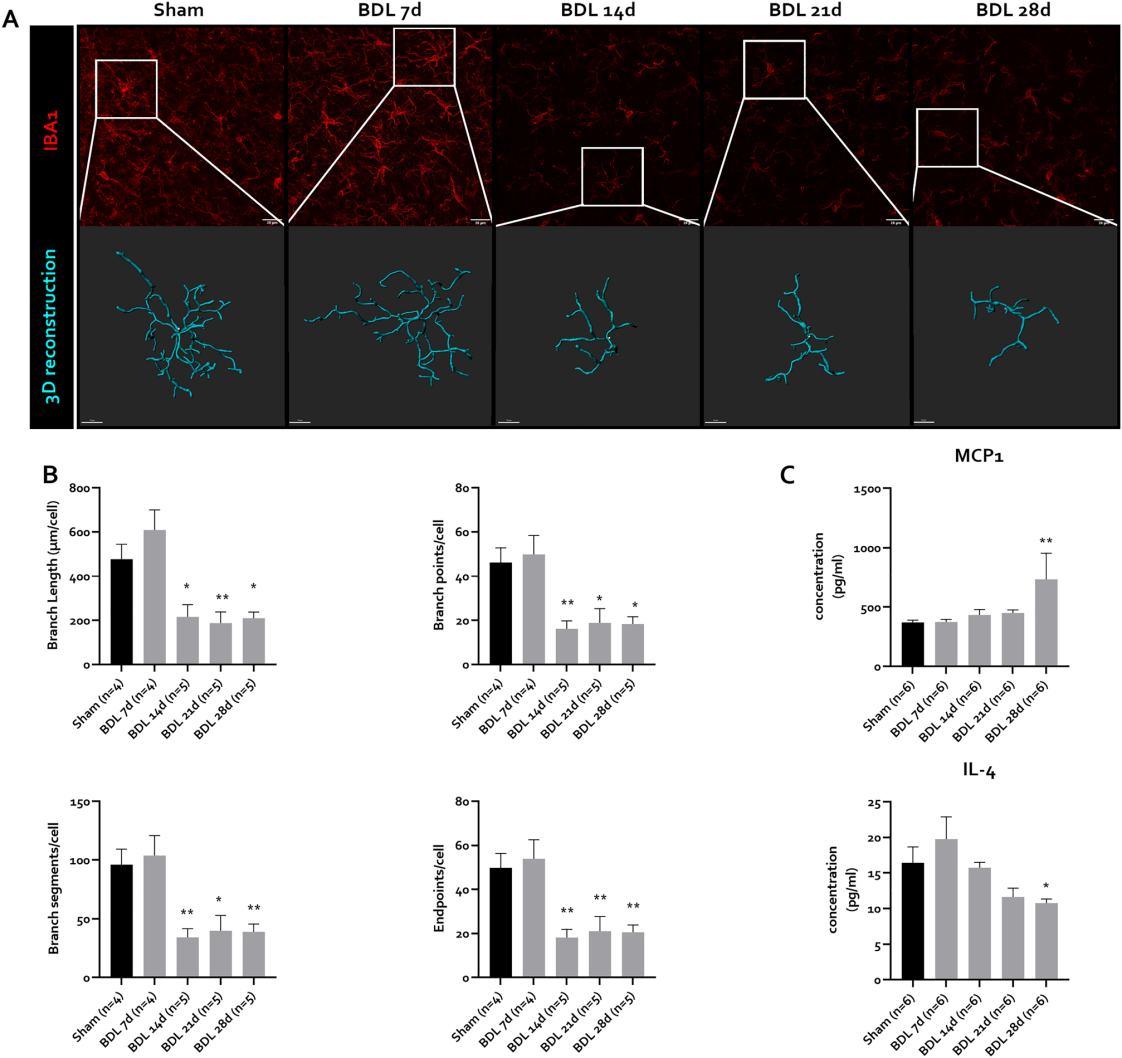
Microglial activation at different timepoints after BDL in C57Bl/6j mice. (**A**) Representative confocal maximum intensity projection images of IBA1 positive microglia and magnified 3D reconstructed microglia in prefrontal cortex at different timepoints after BDL. (**B**) Quantification of 3D reconstructed microglia, showing branch length, number of branch points, branch segments and endpoints per cell in sham (n = 4) or BDL animals (n = 4–5/timepoint) at different timepoints after BDL. (**C**) Protein levels of MCP-1 and IL-4 in prefrontal cortex in sham (n = 6) or BDL animals (n = 6/timepoint) at different timepoints after BDL. *p < 0.05, **p < 0.01. All data are represented as mean ± SEM. Scale bar IBA1 = 20 µm. Scale bar 3D-reconstruction = 10 µm. *BDL* bile duct ligation.

Similar to astrocytes, microglia react to a pathological CNS environment by changing into an activated phenotype. This activated phenotype is characterized by morphological changes, namely a reduction in branching complexity, with fewer and shorter branches^32^. We assessed microglial complexity through 3D reconstruction of allograft inflammatory factor 1 (IBA1) positive cells. A decrease in branch number, length and branch points, as well as terminal points is observed from 14 days onward (Fig. 8A,B), indicating the presence of pathological microglia. Soma size and branch thickness was not obviously altered (Fig. 8A). This altered phenotype coincides with a decrease in the area covered by IBA1 positive cells (Fig. S5B,C), an effect that was consistent over both hippocampus and cortex. Interestingly, microglial activation is not associated with proliferation (Fig. S5B,C) and even a decrease in expression levels of microglial marker *Aif1*, the gene encoding for the IBA1 protein (Fig. S5D). Activated microglia promote the inflammatory environment by altering secretion of cytokines and chemokines^54^. In the cortex, increased protein levels of MCP-1 and decreased levels of IL-4 were detectable 28 days after induction (Fig. 7C). Other cytokines (IL-6, IL-1β, TNF, IL-10, IL-17) and chemokines (MIP-1α, MIP-1β, RANTES) are not altered in BDL mouse brains (Fig. S6).

## Discussion

Currently, the best-characterized animal model in hepatic encephalopathy (HE) research is the bile duct ligation (BDL) rat model. Despite availability of mouse models for advanced chronic liver disease (CLD)^7^, central nervous system (CNS) changes in these models are not well characterized. Here, we are the first to systematically describe the development and temporal evolution of key characteristics of HE in chronic BDL mice. Additionally, we characterize the neurometabolic and gliovascular changes in BDL mouse brains which provides a framework for outcome measures in future studies, but also identifies possible contributing factors in HE development.

First, we show that the criteria for an HE model according to the International Society of Hepatic Encephalopathy and Nitrogen Metabolism (ISHEN) guidelines have been fulfilled^5^. We establish that mouse BDL induces behavioural changes and liver function failure with hyperammonemia. Importantly, hyperammonemia and motor dysfunction is absent in Swiss mice, a strain with known cirrhosis development 6 weeks after BDL^8,9^. Genetic differences likely account for these striking differences in phenotype. Interestingly, one group has reported that C57Bl/6j mice exhibit striking levels of gut barrier failure, gut dysbiosis and bacterial translocation compared to A/J mice. As ammonia is a gut-derived toxin and bacterial translocation is known to provoke HE, this might provide a clue as to the origin of this differential phenotype^46^. Additionally, another group has reported alterations in hepatic ammonia metabolization after BDL in C57Bl/6j mice, while no such observation has been reported in Swiss mice^11^. Careful consideration with regards to strain selection is therefore imperative in future research. Notably, other groups have independently reported hyperammonemia in BDL mice^11,14^, suggesting good reproducibility. Kinetics however differ, with one group describing hyperammonemia as early as 7 days post-induction^11^. Processing differences might explain variable results, as ammonia measurement is highly susceptible to preanalytical differences. Therefore, strict adherence to processing advice recently postulated by ISHEN is essential, as was done in our study^5^. In line with other studies^10–12^, we report that BDL mice reproduce the phenotype of decompensated cirrhosis already at 14 days after induction. Later timepoints are useful for the investigation of long-term effects of chronic HE.

Next, we show that BDL, only in C57Bl/6j mice, induces clear motor function deficits. It should be noted that the motor behavioural changes occur before hyperammonemia and are thus likely affected by non-ammonia related factors. Early systemic inflammation might contribute to these behaviours^55^. However, levels of circulating IL-6 are however only very mildly elevated in C57Bl/6j mice at early timepoints. Moreover, levels of circulating cytokines are comparable between Swiss and C57Bl/6j mice after BDL, while Swiss mice do not develop motor dysfunction. As suggested previously^13,30,31^, influx of inflammatory monocytes into the brain could induce this sickness behaviour in inflammatory liver injury in C57Bl/6j mice. Because of this marked motor dysfunction, reliable cognitive function assessment was not possible. In the future, cognitive tests less affected by motor phenotype, like the passive avoidance test^14^, might be considered to distinguish behavioural deficits related to hyperammonemia and inflammatory liver injury.

With the use of cerebrospinal fluid (CSF) metabolomics, we investigate for the first time the metabolome in CSF of BDL mice. In line with patient data^18^ and BDL rats^19,28^, glutamine levels reproducibly rise secondary to plasma ammonia in BDL mice, consuming glutamate. With regards to other osmolytes, transient decreases in creatine and taurine are detected as possible compensatory osmoregulatory mechanisms. Remarkably, creatine and taurine levels return to baseline in later timepoints, while glutamine levels remain stably elevated. This might suggest that taurine and creatine levels decrease in response to the rapid relative changes in glutamine concentrations, while in later timepoints, a new steady state develops. Alternatively, increased transport into the brain might account for the replenishing of creatine and taurine amounts in the brain^56^.

Energy metabolism dysregulation, through the direct inhibitory effect of ammonia on tricarboxylic acid (TCA) cycle enzymes and/or the preferential allocation of energy towards the activated immune system, has been put forward as an important pathogenic mechanism in HE^4^. Lactate accumulation secondary to energy failure could additionally play a role in brain edema in BDL rats^23^. To assess energy status, we measured levels of ATP and its derivatives ADP and AMP. In our study, AMP depletion in BDL mice could suggest that energy production in the brain is altered. However, since we were unable to detect ATP and ADP, these results should be interpreted with caution. Importantly, ADP and ATP levels have also been found to be decreased in BDL rat brains, suggesting other mechanisms like defective nucleotide synthesis as a possible cause for decreased purine levels^20^. In line with patient data^15^, we could not detect differences in TCA cycle intermediates. Additionally, we could not detect differences in CSF lactate levels, suggesting these mechanisms are not directly involved in mouse HE.

The role of cerebral, ammonia-induced, oxidative stress in HE is controversial^49–51^. In line with previous data^19,20^, we observe no alterations in glutathione-related metabolites in the CSF of BDL mice, suggesting cerebral oxidative stress in BDL mice is limited.

Another finding of interest for future investigation is the accumulation of (tauro-)conjugated bile acids, which has been described in both HE patients^15^ and experimental animals^24^. Interestingly, bile acids accumulate before the development of liver function failure. In a recent study, CNS accumulation of conjugated bile acids before the development of hyperammonemia in a model of non-alcoholic steatohepatitis with hepatocellular carcinoma is associated with astrocyte and neuronal loss, suggesting an independent role for bile acids in the brain^47^. In acute HE models, bile acids have been found to influence neuroinflammatory signaling^48^. In agreement with our data, taurocholic acid has been shown to induce neuronal MCP-1 signaling through sphingosine-1-phosphate receptor 2^57^.

In decompensated cirrhosis, tryptophan metabolization through the tryptophan–kynurenine pathway is amplified^25^. Surprisingly, but in line with patient data^15^, we find increased levels of tryptophan in CSF in BDL mice, already 7 days after induction. Possible explanations include a higher free tryptophan concentration, which can pass the blood–brain barrier (BBB), due to hypoalbuminemia^25^. Also, active transport of tryptophan over the BBB is in competition with branched chain amino acids, which are depleted in cirrhosis^58^. Interestingly, we also detect kynurenine, a marker of neuroinflammation^26^, from 14 days onward, the timepoint where we detected microglial morphological changes consistent with neuroinflammation. As targeting the kynurenine pathway has proven beneficial in preclinical models of depression, this could prove to be an interesting therapeutical target for future investigation^59^.

In short, mouse BDL in C57/Bl6j mice presents with a CSF metabolic profile consistent with HE. Our data do not support a significant contribution of CNS energy metabolism dysfunction or oxidative stress, while cerebral bile acid signaling and the role of the tryptophan–kynurenine pathway in mouse HE deserve further investigation, with special focus on their role in neuroinflammation. As the CSF is an approximator of brain metabolic status, future studies should investigate whether these metabolic changes are present in brain tissue. As brain regions have been found to react differently in HE, investigation of other brain regions like the cerebellum might uncover similar regional variability in this model.

Finally, we are the first to perform extensive morphological and longitudinal evaluations of glial cells in BDL mice. With regards to astrocytes, alterations in glutamine already suggest their involvement in HE mice. For the first time, we report time-dependent morphological and molecular alterations in astrocytes in BDL mice. Astrocytes are instrumental in BBB homeostasis^60^. We have established that BBB permeability is altered, suggesting that astrocyte morphological alterations go together with pathological BBB perturbation^52^. The fact that clear changes in gene expression and protein subcellular redistributions of tight junction components seem to be absent suggests there is no major structural breakdown of the BBB in BDL mice. As suggested in BDL rats, functional BBB breakdown through decreased astrocytic vessel coverage might be the operant mechanism^24^.

We also observe activated microglial morphology as soon as 14 days after BDL induction. This is likely to be caused, at least in part, by systemic inflammation^61,62^ rather than ammonia alone^63^. It should be noted that accompanying soma enlargement and thickening of processes seen in classically activated microglia^32^ is not obvious in microglia after BDL, possibly indicating a transitional state of these microglia. Alternatively, senescent microglia can present as deramified cells with retracted processes^64^ and might account for the specific morphological phenotype observed in these animals. Interestingly, ammonia has been found to induce senescence in astrocytes and senescence markers have been detected in brain tissue of HE^ 65,66^. However, whether ammonia with or without systemic inflammation similarly affects microglia in HE remains unknown.

Strikingly, only limited changes in CNS cytokines are detectable despite obvious morphological changes in microglial cells. However, this is in line with previous data on the effects of chronic low-grade inflammation on brain tissue cytokine levels^62^. Most likely, cyto/chemokine changes are relatively subtle and therefore lost due to bulk assessment of all neuronal cell types, as has been reported before in aged microglia^67^. Alternatively, microglia may present with a chronic effector phenotype, characterized by low cytokine release^68^. More specific investigation of enriched microglial populations in our model might provide the answer in the future.

Surprisingly, microglial activation is associated with a downregulation of *Aif1*, the gene encoding for allograft inflammatory factor 1 (IBA1), transcription in BDL mouse brains. Similar results were reported in sorted microglia after LPS injection^69^ as well as in disease associated microglia in Alzheimer’s disease^70^, suggesting the often reported increased *Aif1* expression in neuroinflammation is rather due to microglial proliferation than activation. Accordingly, microglia do not proliferate in BDL mice, confirming earlier data^13^. Interestingly, as senescent cells are unable to divide due to cell cycle arrest^71^, a senescent phenotype would possibly account for this absence in proliferation.

From the presented data, it is evident that glial cell function is altered in HE. As this study is descriptive in nature, whether these changes are causal or rather secondary to the observed phenotype remains unclear. Future research should focus on determining the role of glial cell dysfunction in behavioural HE phenotype, as well as on the molecular mechanisms underpinning astrocyte reactivity and microglial activation and how these cell types interact in BDL mice. Based on our data, the contribution of hyperammonemia, (systemic) inflammation and bile acid accumulation in the development of glial cell dysfunction are obvious points of future research. Additionally, it has become increasingly clear that astrocytes^72^ and microglia^73^ are very heterogeneous, and efforts should be undertaken to assess the role of different types of astrocytes in HE.

In conclusion, we show that mouse BDL in C57Bl/6j mice can be used as a model for HE in CLD. This model has the benefit of possible genetic manipulation, which will allow research into CNS specific changes in HE, a drawback of the current most widely used model, the rat BDL model^5^. It however also has disadvantages compared to the rat BDL model, most prominently the confounding sickness behaviour that develops before the development of hyperammonemia. In future studies, depending on the research question, it is advisable to consider advantages and disadvantages of each rodent species. While BDL rats remain ideally suited for e.g. the study of the effects of ammonia-lowering compounds^5,74^, BDL mice provide a valuable tool for fundamental research into the brain-specific pathophysiology of HE. Additionally, we identify several mechanisms possibly involved in disease development in this model, including bile acid accumulation, tryptophan–kynurenine pathway activation and (neuro-)inflammation. This warrants further research into their contribution to the HE phenotype, as well as the therapeutic potential of targeting these pathways in clinical HE.

## Supplementary Information

Supplementary Information.

## Data Availability

The data generated during the current study are available from the corresponding author upon reasonable request.

## Abbreviations

BBB: Blood–brain barrier
BDL: Bile duct ligation
CLD: Chronic liver disease
CNS: Central nervous system
CP: Ceruloplasmin
CSF: Cerebrospinal fluid
FKBP5: FK506 binding protein
GFAP: Glial fibrillary acidic protein
GIM: Goat immunomix
HE: Hepatic encephalopathy
IBA1: Allograft inflammatory factor 1
ISHEN: International Society of Hepatic Encephalopathy and Nitrogen Metabolism
NGVU: Neurogliovascular unit
NOR: Novel object recognition
OCLN: Occluding
OFT: Open field test
RT: Room temperature
SERPINA3N: Serpin family A member 3
TCA: Tricarboxylic acid
ZO-1: Zonula occludens-1
