# Supplementary material for: A mouse model of hepatic encephalopathy: bile duct ligation induces brain ammonia overload, glial cell activation and neuroinflammation
Source: Sci Rep. 2022 Oct 20;12:17558. doi: 10.1038/s41598-022-22423-6 (PMC9585018; doi:10.1038/s41598-022-22423-6)
Supplement: Supplementary file 1 — Supplementary Information. [file 41598_2022_22423_MOESM1_ESM.docx]

**Supplemental material and methods**

Neurobehaviour assessment

As a measure of balance, difficult beam traversal test was performed according to [1]. Two clean mouse cages were inverted on a tabletop and a beam of 1 m length consisting of four sections with narrowing widths (3.5, 2.5, 1.5, and 0.5 cm) was placed on top of the inverted mouse cages. The home cage of the mice was placed at the end of the beam so that the narrowest end of the beam led right into the home cage. The mice were picked up by the base of their tails and placed at the wide end of the beam. The time the mice needed to traverse the beam was recorded. For training trials (2 days, 5 trials per mouse), no grid was placed on the beam. For the testing trials (five in total per mouse at each timepoint), a mesh grid that corresponded to each beam width was placed on top of the beam. The mesh grid had 1 cm squares and left a 1 cm space between the grid and the beam surface. The mean of the 3 best testing trials at each timepoint was used for analysis.

For assessment of general motor function and anxiety, an open field test (OFT) was performed. Mice were placed in a rectangular, clear open-field area made from acrylic (40 × 40 × 40 cm) and were allowed to move freely for 5 min. All movement was recorded. Total exploration time was assessed as an outcome measure for general motor function and time spent in the center was assessed as a measure of anxiety.

For assessment of short term memory a novel object recognition (NOR) test was performed, adapted from [2]. Mice were placed in the aforementioned acrylic open field. The procedure includes three phases: habituation, training, and testing. NOR testing was performed 24 h after OFT, which served as a habituation phase. In the training phase, mice were placed in the arena facing the wall opposite to two identical objects which were put at two opposite positions in the box at the same distance from the nearest corner. Mice were allowed to freely explore the two objects for 5 min and were then returned to their home cage. In the testing phase, memory was tested 15 min after the training session to evaluate short term memory. Mice were placed back in the same box, where one familiar object was replaced by a novel object. The exploration of the objects was considered as deliberate contact with their mouth or nose that occurred with each object. The exploration time for the familiar or the new object during the test phase was recorded. The percentage preference for the novel object was calculated using the following formula: % novel object preference = [novel object exploration time/(novel object exploration time + familiar object exploration time)] × 100%. A value of 50% reflects no preference for any of the objects, and a value > 50% indicates preference for the novel object. Mice exhibiting too little explorative behaviour, i.e. a total exploration time of <5 seconds were excluded from further analysis.

As a measure of spatial memory, a T-maze test was performed. Mice were placed at the base arm of a T-maze (arms 45 x 6 x 4 cm, placed on platform of 23 cm height). The mice were left free to explore until a goal arm was chosen. The opposite arm was closed off and mice were allowed to move for 5 min in the chosen arm and the base arm. Immediately afterwards, the guillotine closing off the novel arm was opened and the mouse was placed in the base arm. Mice were allowed to explore the T-maze freely, and time spent in the known and novel arm was recorded. Preference for the novel arm was determined using the following formula: % novel arm preference = novel arm exploration time/total exploration time. A value of >33% indicates a preference for the unexplored arm. Mice exhibiting too little explorative behaviour, i.e. total arm entries <4, were excluded from further analysis.

**Supplementary references**

[1] S. M. Fleming, O. R. Ekhator, and V. Ghisays, “Assessment of Sensorimotor Function in Mouse Models of Parkinson’s Disease,” *J. Vis. Exp.*, no. 76, p. 50303, Jun. 2013, doi: 10.3791/50303.

[2] S. M. Hölter *et al.*, “Assessing Cognition in Mice,” *Curr. Protoc. Mouse Biol.*, vol. 5, no. 4, pp. 331–358, Dec. 2015, doi: 10.1002/9780470942390.mo150068.

**
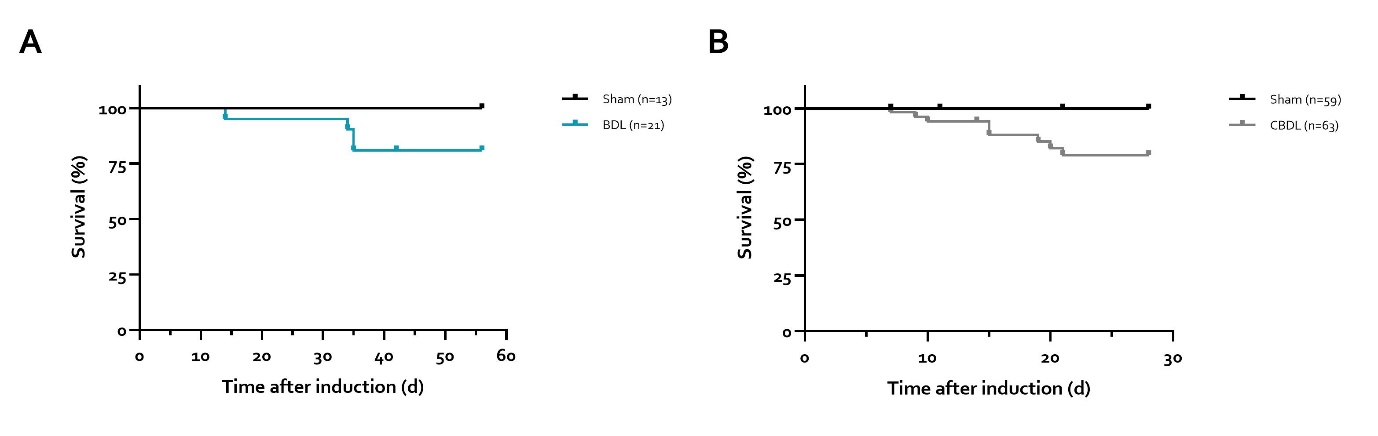
**

**Figure S1. Overall survival after BDL surgery in (A) Swiss and (B) C57Bl/6j mice**.

**Figure S2. Ammonia and osmolytes in cerebrospinal fluid after chronic BDL in C57Bl/6j mice. A-B**. Relative (**A**) glutamine, glutamate, (**B**) taurine and creatine levels in CSF of sham (n=4) and BDL (n=4) animals, 28 days after induction. Values are represented as mean ± SEM. * p < 0.05. BDL, bile duct ligation; CSF, cerebrospinal fluid.

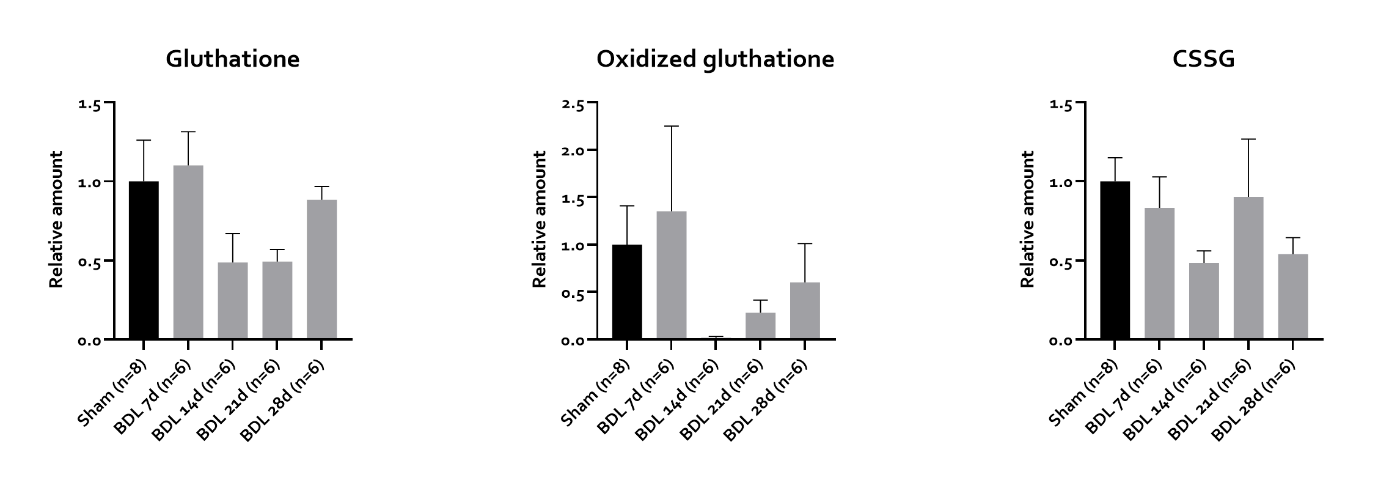
**Figure S3. Redox molecules in cerebrospinal fluid post BDL in C57Bl/6j mice.** Relative levels of gluthatione, oxidized gluthatione and CSSG in CSF of sham (n=8) controls and BDL (n=6/timepoint) mice at different timepoints after induction. Values are represented as mean ± SEM. BDL, Bile duct ligation; CSF, Cerebrospinal fluid


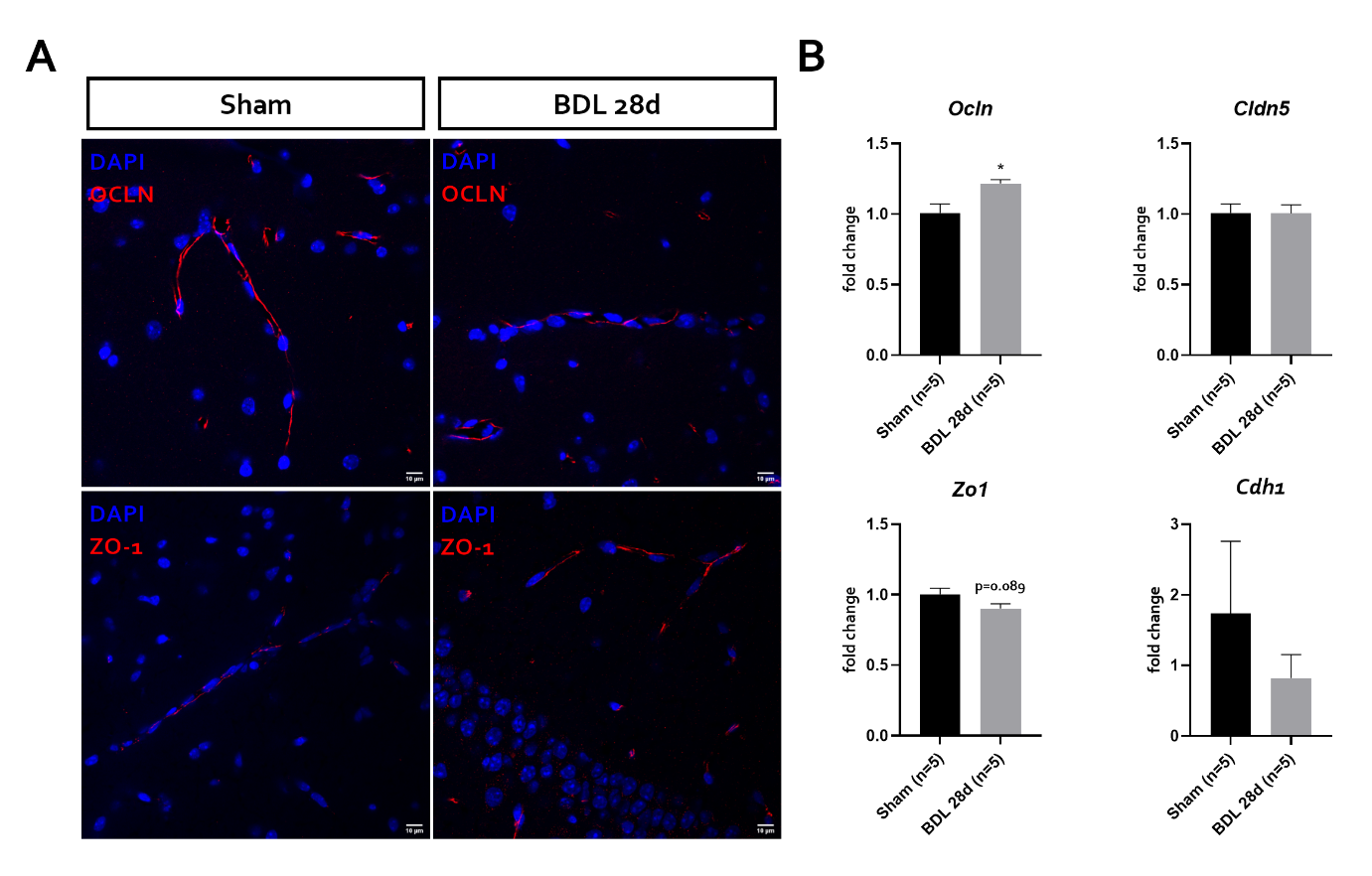
**Figure S4. Characterization of tight junctions at the blood-brain barrier (BBB) after BDL in C57Bl/6j mice. A**. Representative confocal images of TJ proteins OCLN and ZO-1 in the hippocampus. **B.** Expression levels of Ocln, Zo1, Cldn5 and Cdh in the hippocampus in sham (n=5) and BDL (n=5) mice, 28 days after induction. * p < 0.05. All data are represented as mean ± SEM. Scale bar = 10 μm. BDL, bile duct ligation; Cdh1, E-Cadherin; CLDN5, claudin-5; OCLN, occludin; TJ, tight junction; ZO-1, zonula occludens.

**
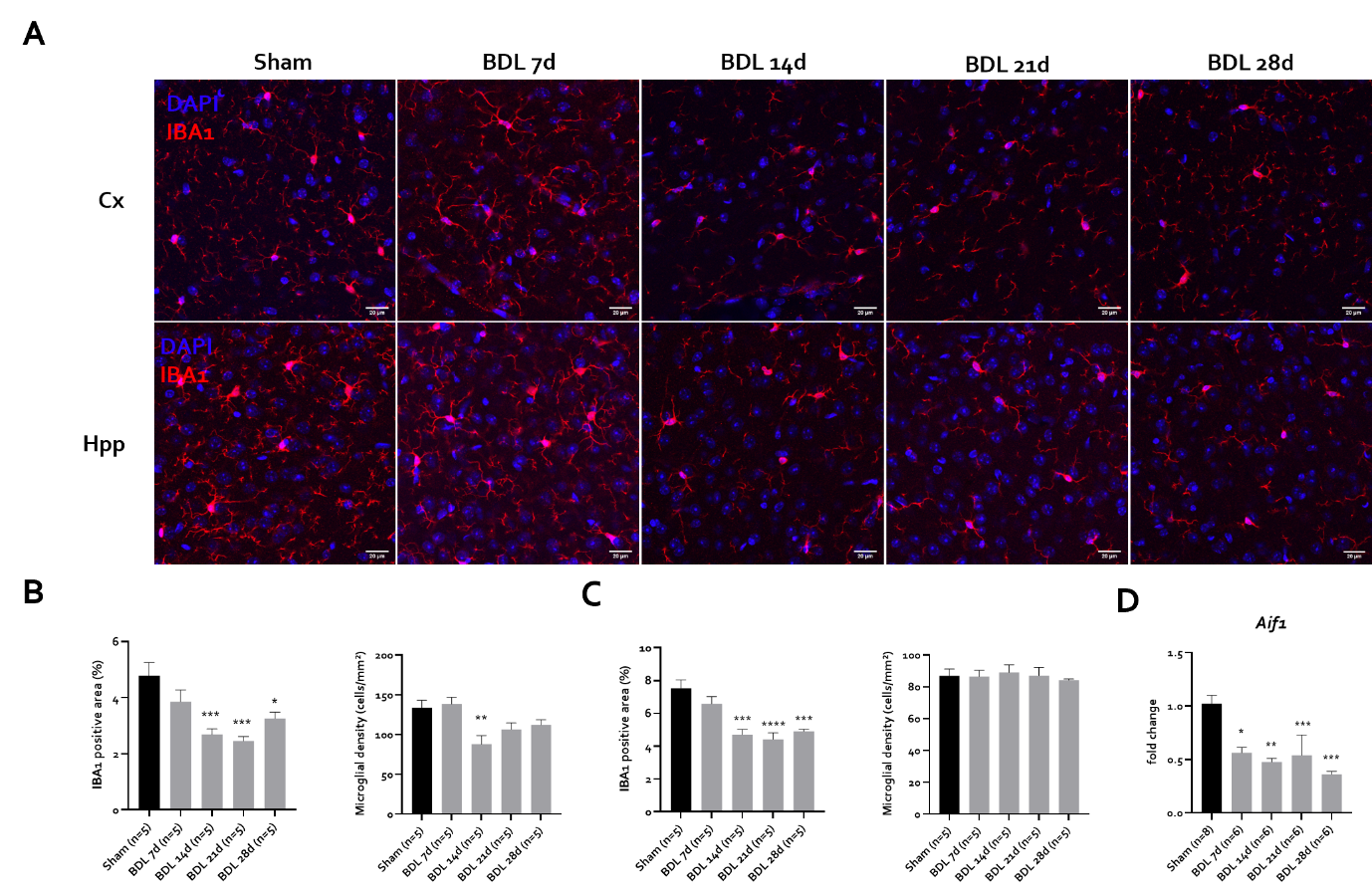
Figure S5. Microglial coverage, density and marker expression after chronic BDL in C57Bl/6j mice. A.** Representative images of IBA1+ cells in cortex and hippocampus of sham and BDL mice at different timepoints. **B.** IBA1 positive area and microglial count in cortex of sham (n=5) and BDL (n=5/timepoint) mice at different timepoints. **C.** IBA1 positive area and microglial count in hippocampus of sham (n=5) and BDL (n=5/timepoint) mice at different timepoints**.** **D.** Expression levels of microglial marker Aif1 in sham and BDL mice at different timepoints. * p < 0.05, ** p < 0.01, *** p < 0.001. All data are represented as mean ± SEM. Aif1, allograft inflammatory factor 1; BDL, bile duct ligation; Cx, cortex; Hpp, hippocampus; Iba1, allograft inflammatory factor 1.

**
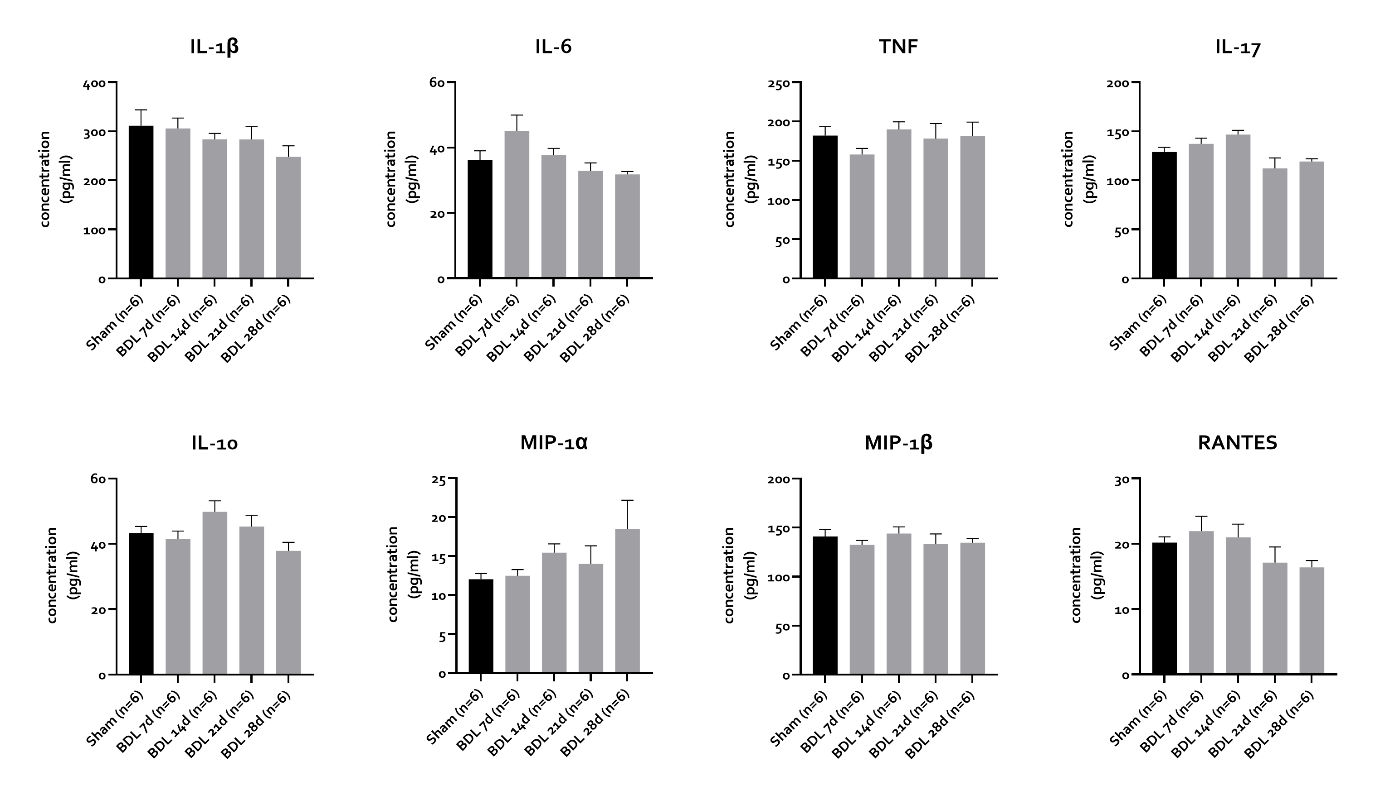
Figure S6. Cytokine and chemokine levels after BDL in C57Bl/6j mice.** Protein levels of pro-inflammatory cytokines (IL-1β, IL-6, TNF, IL-17), anti-inflammatory cytokines (IL-10) and chemokines (MIP-1α, MIP-1β, RANTES) in prefrontal cortex of sham (n=6) and BDL (n=6/timepoint mice at different timepoints after induction. All data are represented as mean ± SEM. BDL, bile duct ligation; IL, interleukin; MIP, macrophage inflammatory protein; RANTES, regulated on activation normal T cell expressed and secreted TNF, tumor necrosis factor.

**Supplementary Table 1. List of antibodies used**

| **Primary antibody** | **Secondary antibody** | **Section type** | **Application** |
| --- | --- | --- | --- |
| Rabbit anti-IBA1  (019-19741, Wako; 1/500) | Alexa Fluor-633 goat anti-rabbit (A21070, Thermo Scientific, 1:400) | 50 μm vibratome  5 µm paraffin section | Detection of microglia |
| Rabbit anti-GFAP (1/1000, Agilent, Z033429-2) |  | 50 μm vibratome | Detection of astrocytes |
| Rabbit anti-ZO-1  (617300, Invitrogen; 1/500) |  | 20 μm cryosection | Detection of tight junction proteins |
| Mouse anti-OCLN  (33-1500, Invitrogen; 1/100) | Alexa Fluor-633 goat anti-mouse (A21052, Thermo Scientific, 1:400) | 5 μm paraffin section |  |

**Supplementary Table 2. List of primer sequences used for RT-qPCR analysis.**

| **Gene** | **Fw Primer (5’->3’)** | **Rev Primer (5’->3’)** |
| --- | --- | --- |
| *Aif1* | ATCAACAAGCAATTCCTCGATGA | CAGCATTCGCTTCAAGGACATA |
| *Fkbp5* | TGAGGGCACCAGTAACAATGG | CAACATCCCTTTGTAGTGGACAT |
| *Cp* | CTTAGCCTTGGCAAGAGATAAGC | GGCCTAAAAACCCTAGCCAGG |
| *Serpina3n* | ATTTGTCCCAATGTCTGCGAA | TGGCTATCTTGGCTATAAAGGGG |
| *Zo1* | AGGACACCAAAGCATGTGAG | GGCATTCCTGCTGGTTACA |
| *Cldn5* | GCAAGGTGTATGAATCTGTGCT | GTCAAGGTAACAAAGAGTGCCA |
| *Ocln* | CCAGGCAGCGTGTTCCT | TTCTAAATAACAGTCACCTGAGGGC |
| *Cdh1* | TCGGAAGACTCCCGATTCAAA | CGGACGAGGAAACTGGTCTC |
| *Hprt* | AGTGTTGGATACAGGCCAGAC | CGTGATTCAAATCCCTGAAGT |
| *Rpl* | CCTGCTGCTCTCAAGGTT | TGGTTGTCACTGCCTGGTACTT |
| *Ubc* | CTTTCCAGAGAGCGGAACAG | CAAGAACTGCGACCCAAATC |
| *Gapdh* | TGAAGCAGGCATCTGAGGG | CGAAGGTGGAAGAGTGGGAG |
| *B2m* | ATGCACGCAGAAAGAAATAGCAA | AGCTATCTAGGATATTTCCAATTTTTGAA |
